# Critical Evaluation of Acetylcholine Determination in Rat Brain Microdialysates using Ion-Pair Liquid Chromatography with Amperometric Detection

**DOI:** 10.3390/s8085171

**Published:** 2008-08-28

**Authors:** Dimitri De Bundel, Sophie Sarre, Ann Van Eeckhaut, Ilse Smolders, Yvette Michotte

**Affiliations:** Experimental Neuropharmacology Research Group, Department of Pharmaceutical Chemistry and Drug Analysis, Vrije Universiteit Brussel, Laarbeeklaan 103, 1090 Brussel, Belgium; E-Mails: ddebunde@vub.ac.be (D.D.B.); Sophie.Sarre@vub.ac.be (S.S.) ; aveeckha@vub.ac.be (A.V.E.); Ilse.Smolders@vub.ac.be (I.S.)

**Keywords:** Acetylcholine, microdialysis, liquid chromatography, amperometric detection

## Abstract

Liquid chromatography with amperometric detection remains the most widely used method for acetylcholine quantification in microdialysis samples. Separation of acetylcholine from choline and other matrix components on a microbore chromatographic column (1 mm internal diameter), conversion of acetylcholine in an immobilized enzyme reactor and detection of the produced hydrogen peroxide on a horseradish peroxidase redox polymer coated glassy carbon electrode, achieves sufficient sensitivity for acetylcholine quantification in rat brain microdialysates. However, a thourough validation within the concentration range required for this application has not been carried out before. Furthermore, a rapid degradation of the chromatographic columns and enzyme systems have been reported. In the present study an ion-pair liquid chromatography assay with amperometric detection was validated and its long-term stability evaluated. Working at pH 6.5 dramatically increased chromatographic stability without a loss in sensitivity compared to higher pH values. The lower limit of quantification of the method was 0.3 nM. At this concentration the repeatability was 15.7%, the inter-day precision 8.7% and the accuracy 103.6%. The chromatographic column was stable over 4 months, the immobilized enzyme reactor up to 2-3 months and the enzyme coating of the amperometric detector up to 1-2 months. The concentration of acetylcholine in 30 μl microdialysates obtained under basal conditions from the hippocampus of freely moving rats was 0.40 ± 0.12 nM (mean ± SD, n = 30). The present method is therefore suitable for acetylcholine determination in rat brain microdialysates.

## 1. Introduction

Acetylcholine is a prominent neurotransmitter of the peripheral and the central nervous system. In the central nervous system, acetylcholine is involved in attention, learning, memory, consciousness, sleep, and control of voluntary movements [1-5]. Dysfunction of the cholinergic system is implicated in major neurological disorders such as schizophrenia, Alzheimer's disease, Parkinson's disease and Huntington's disease [6]. Acetylcholine is formed from its precursors choline and acetyl coenzyme A by choline acetyltransferase and released from cholinergic nerve terminals into the synaptic cleft between presynaptic and postsynaptic neurons [7]. The resulting chemical signal conveyed by acetylcholine is terminated by its enzymatic degradation. Acetylcholine is rapidly metabolized to acetate and choline by acetylcholinesterase, but a small fraction leaks out of the synaptic cleft into the extracellular fluid [7]. As such, the extracellular concentration of acetylcholine can be measured to assess the activity of cholinergic neurons.

The *in vivo* microdialysis technique is extensively used to monitor the concentration of acetylcholine in the extracellular milieu of designated brain regions in conscious animals [8]. This technique has brought an important contribution to the current understanding of the physiological role of acetylcholine and its involvement in pathological conditions. A probe with a semi-permeable dialysis membrane is implanted into the brain area of interest and slowly perfused with a physiological solution. Small water soluble molecules migrate through the pores of the microdialysis membrane by passive diffusion, creating a dynamic equilibrium between the extracellular fluid and the perfusion liquid [9]. Typically, 5-50 μL samples are collected at a flow rate of 0.5-2.5 μL/min. Depending on the perfusion rate, location of the probe, the timing of implantation, the composition of the perfusion liquid, dimensions of the membrane and its diffusion properties, acetylcholine concentrations in basal microdialysis samples range between 0.1-3 nM [10-16]. The application of the microdialysis technique for intracerebral acetylcholine monitoring therefore requires a highly sensitive and reliable method for acetylcholine quantification.

**Figure 1.**
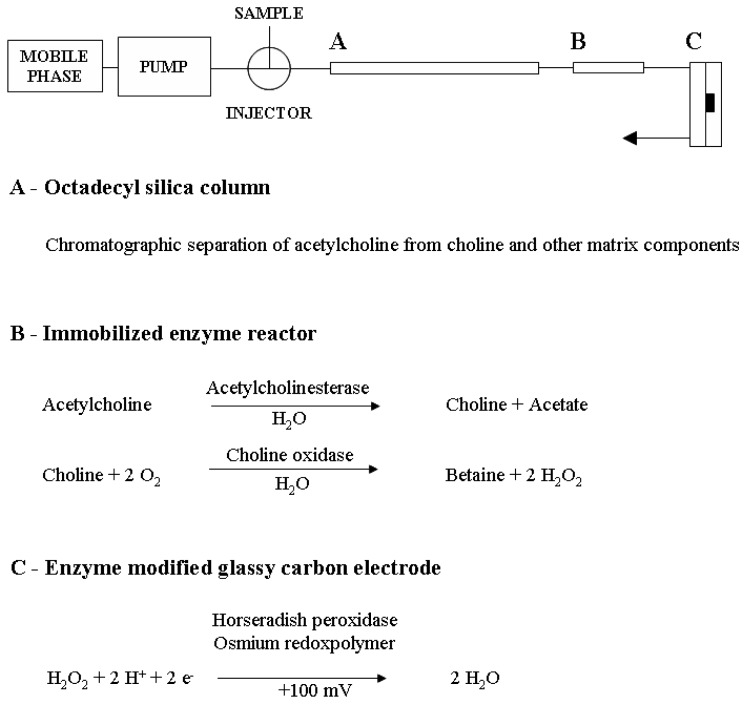
Chromatography setup.

Liquid chromatography with tandem mass spectrometry was more recently developed as a sensitive but expensive method for acetylcholine determination in microdialysis samples, reaching a detection limit of 0.02-0.03 nM [16-19]. However, the most widely used method for acetylcholine quantification in brain microdialysis samples to date remains liquid chromatography with electrochemical detection. This method is based on the chromatographic separation of acetylcholine from choline and other matrix components, conversion of acetylcholine to hydrogen peroxide by acetylcholine esterase and choline oxidase and amperometric detection of the produced hydrogen peroxide [20]. A detection limit of 0.2-2 nM was reported for microbore liquid chromatography systems with acetylcholine esterase and choline oxidase chemically bound in an immobilized enzyme reactor (IMER) and hydrogen peroxide detection on a horseradish peroxidase osmium-redox polymer coated glassy carbon or gold ring disc electrode (Figure 1) [10-15, 21-23]. This assay is sufficiently sensitive to detect acetylcholine in basal microdialysis samples under optimized conditions, but has not been thoroughly validated within the corresponding concentration range. Furthermore, a limited robustness of the chromatographic separation and enzymatic conversions has been reported [22, 24]. Consequently, in most microdialysis studies a cholinesterase inhibitor, such as neostigmine or physostigmine, is added to the perfusion liquid to augment the concentration of acetylcholine in the extracellular fluid and facilitate reliable quantification [25]. However, artificially increasing acetylcholine concentrations exerts a significant influence on the cholinergic system and may confound the interpretation of drug effects or mask alterations in acetylcholine transmission in animal models for pathology [25-27]. Further optimization of the chromatographic parameters may therefore lead to a more reliable determination of acetylcholine in microdialysis samples without the use of cholinesterase inhibitors.

Two liquid chromatographic methods have been combined with the amperometric determination of acetylcholine: ion-pair chromatography with octadecyl silica columns as the stationary phase [28] and ion-exchange chromatography with kation-exchange resin columns as the stationary phase [29, 30]. The composition of the mobile phase is determined by the requirements for optimal activity and stability of the used enzyme systems: essentially an aqueous phosphate buffer at pH 6.5-8.5 [28-31]. The conversion of acetylcholine to hydrogen peroxide is most efficient at pH 8.0-8.5 [29, 30]. However, the optimal pH for the subsequent detection of hydrogen peroxide on the enzyme modified electrode has not been determined. The cation-exchange setup provides good chromatographic stability under these conditions, but a limited resolution between acetylcholine and choline [12, 29, 30]. Microdialysis samples typically contain a high concentration of choline, which may interfere with the acetylcholine signal. A precolumn choline oxidase and catalase reactor was developed to eliminate choline from the microdialysis sample matrix [13, 21]. The ion-pair setup offers a superior resolution between acetylcholine and choline and does not require the preliminary elimination of choline [23, 31]. However, the stability of silica-based chromatographic columns is highly dependent on the mobile phase pH [32]. The feasibility of acetylcholine determination in an ion-pair chromatographic setup with amperometric detection was previously reported at pH 6.5, but the implications of lowering the mobile phase pH for the sensitivity and long-term enzymatic and chromatographic stability were not investigated [23].

In the present study an ion-pair liquid chromatography method with amperometric detection was optimized and validated for acetylcholine determination in microdialysis samples. Different mobile phase conditions were compared to improve the sensitivity, long-term enzymatic and chromatographic stability. The polymer-coated octadecyl silica column type used in this study allowed a reliable separation of acetylcholine from choline and other matrix components over 4 months of intensive use.

## 2. Results and Discussion

### 2.1. Method optimization

**Figure 2.**
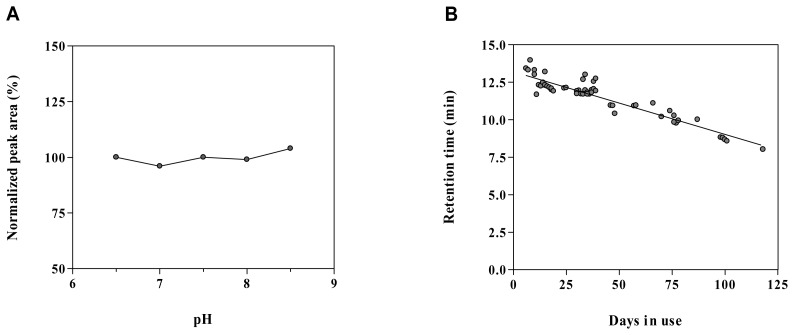
**(A)** Normalized response for acetylcholine (10 nM) as a function of mobile phase pH. **(B)** Column stability at pH 6.5 expressed as the loss in acetylcholine retention as a function of time. Pooled data obtained with three columns at pH 6.5.

The chromatographic parameters were chosen to obtain an optimal equilibrium between sensitivity and chromatographic stability. A microbore polymer-coated silica column with high endcapping and low octadecyl binding-density was selected to allow chromatographic stability when using a purely aqueous phosphate buffer as the mobile phase. Typically, mobile phases with pH 8.0-8.5 have been used for detection of acetylcholine by liquid chromatography with amperometric detection [10-15]. Nevertheless, it has been demonstrated that it is feasible to detect acetylcholine in microdialysis samples when working at pH 6.5 [23]. In the present setup, no difference in sensitivity was observed within the pH range 6.5-8.5 (Figure 2A).

Given that the stability of silica-based chromatographic columns is dramatically improved at lower pH [32], further development was performed with a mobile phase at pH 6.5. At this pH the chromatographic column was stable over 4 months of intensive use (Figure 2B).

The efficacy of acetylcholine detection can be improved by reducing the flow rate and increasing the ionic strength of the mobile phase [29-31, 33]. However, reduction of the flow rate is limited by the minimal system pressure required for optimal pulse-dampening and corresponding baseline stability for a given chromatographic column [34]. The increase of ionic strength is limited by the silica-column packing degradation observed at higher phosphate concentrations [35]. In the present study, the flow rate was 0.075 ml/min and the phosphate concentration 50 mM.

Reversed-phase ion-pair chromatographic separation of quaternary ammonium compounds, such as choline and acetylcholine, is based on the partition of the ion-pairs formed with anionic counter ions, such as 1-hexanesulphonate or 1-octanesulphonate, between the apolar stationary phase and the polar mobile phase.

Interactions between unpaired choline and acetylcholine with surface silanol groups of silica-based columns may result in strong adsorption of both analytes to the stationary phase, producing irregular retention times and band-broadening or peak-splitting [31]. This can be reduced by adding silanol blocking agents, such as tetramethylammonium or tetraethylammonium salts, to the mobile phase [31, 36]. Excellent chromatographic separation of acetylcholine and choline was previously reported using a 5 mM sodium 1-hexanesulphonate and 4 mM tetramethylammonium chloride on a 2.1×250 mm octadecyl silica column [31].

**Figure 3.**
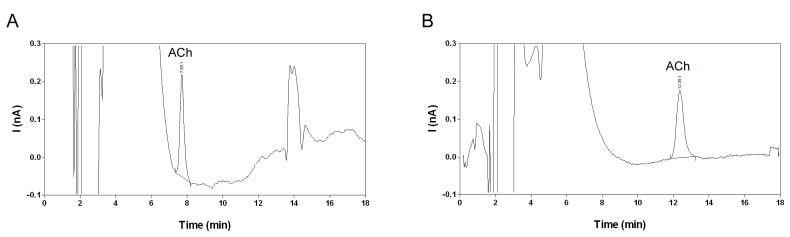
Chromatograms obtained for a 10 nM acetylcholine standard in water using a mobile phase containing **(A)** 5 mM sodium 1-hexanesulphonate and 4 mM tetramethylammonium bromide or **(B)** 1 mM sodium 1-octanesulphonate and 2 mM tetramethylammonium bromide. Other conditions as described in the experimental section. ACh: acetylcholine peak.

**Figure 4.**
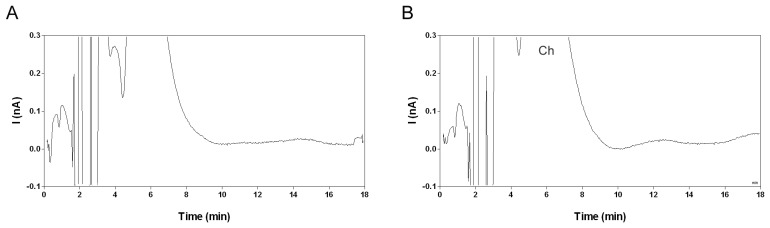

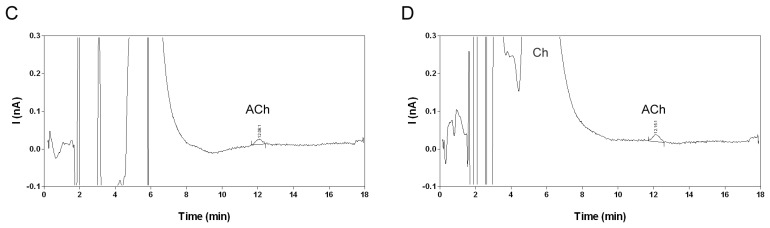
Chromatograms obtained for **(A)** a blank calibration standard in water, **(B)** a 100 nM choline standard in water, **(C)** a 0.3 nM acetylcholine standard in water and **(D)** a hippocampal microdialysis sample. Chromatography and microdialysis conditions as described in the experimental section. ACh: acetylcholine peak. Ch: choline peak, ACh: acetylcholine peak.

In the present study, the aforementioned mobile phase did not yield a statisfactory baseline resolution of acetylcholine from the injection peak on a 1.0×150 mm octadecyl silica column (Figure 3A). This illustrates that ion-pair chromatographic separation can vary greatly depending on the type of column [31]. A complete separation of acetylcholine from the injection peak was obtained with 1 mM 1-sodiumoctanesulphonate and 2 mM tetramethylammonium bromide (Figure 3B). However, choline was not resolved from the injection peak under these chromatographic conditions (Figure 4B). The acetylcholine peak area was not influenced by the concentration of the ion-pairing agent or the silanol blocking agent and was therefore further used as a measure for the acetylcholine concentration.

### 2.2. Selectivity and limit of quantification

The chromatographic separation of acetylcholine from choline and other matrix components, the selective conversion of acetylcholine into hydrogen peroxide by the post-column IMER and the low working potential of the enzyme-coated glassy carbon electrode contribute to the high selectivity of the described method. Acetylcholine calibration samples in water yielded a peak with stable peak area, retention time and signal-to-noise (S/N) ratio. The acetylcholine peak could not be detected in the corresponding blanks (Figure 4A). Choline eluted within the injection peak and could not be quantified (Figure 4B). In fresh microdialysis samples a peak corresponding to acetylcholine was readily detected (Figure 4D). The detection limit estimated as the lowest concentration yielding an S/N ratio of 3 was 0.15-0.2 nM. A similar detection limit was previously described for liquid chromatography methods with amperometric detection [10, 14].

However, the reliability of acetylcholine determination at low concentration levels has hitherto not been investigated. Therefore, the lower limit of quantification (LLOQ) in water was estimated at 0.3 nM for an S/N ratio of 5 (Figure 4C). The LLOQ is defined as the lowest concentration for which the variation on replicate calibration standards is less than 20% and the estimated concentration is within 80-120% of the nominal concentration according to the FDA guideline for bioanalytical method validation [37]. The compliance of the proposed LLOQ with this definition was further evaluated in microdialysis matrix (Section 2.4).

### 2.3. Linearity and matrix effects

Calibration standards and quality control samples spiked with 0.0, 0.3, 1.0, 3.0 and 10.0 nM acetylcholine were prepared in water and microdialysis matrix respectively. The calibration standards were analyzed within one day and peak areas were fitted to a linear regression equation of the format *y* = *ax* + *b*, where *y* represents peak area and *x* represents the concentration of acetylcholine. The mean ± SD equations of the calibration curves (n = 5) were *y* = (56.2 ± 0.5)*x* + (5.0 ± 2.2) and *y* = (55.3 ± 0.6)*x* + (4.9 ± 2.7) for calibration standards prepared in water and quality control samples in microdialysis matrix respectively. The corresponding mean correlation coefficients were 0.9975 and 0.9984. An analysis of variance lack of fit test showed that the calibration curves were linear over the investigated concentration range in water (p=0.59, F_4,20_=0.72) and in microdialysis matrix (p=0.71, F_4,20_=0.53). A two-way analysis of variance showed that the slopes were not significantly different (p=0.82, F_4,32_=0.38). The absence of matrix effects shows that the acetylcholine concentration in microdialysis samples can be effectively determined by interpolation on a linear regression curve prepared in water.

### 2.4. Precision and accuracy

The precision and the accuracy of the present method were determined over three consecutive days. Each day five replicate quality control samples, prepared by spiking 0.0, 0.3, 1.0, 3.0 and 10.0 nM acetylcholine in microdialysis matrix, were analyzed for acetylcholine content through interpolation on a calibration curve obtained with calibration standards prepared in water. The relative standard deviation of the calculated concentrations was used as the index of precision. The accuracy of the method was determined by comparing the calculated concentrations with their nominal values. At the LLOQ level the repeatability and inter-day precision were less than 20% and the accuracy within 80-120%. At all other levels the repeatability and inter-day precision were less than 15% and the accuracy within 85-115%. This demonstrates that the described method is precise and accurate for determination of acetylcholine in microdialysis samples within the range 0.3 – 10.0 nM with 0.3 nM the LLOQ, as defined by the FDA guideline for bioanalytical validation [37]. The results are summarized in Table 1.

### 2.5. Stability in water and microdialysis matrix

**Figure 5.**
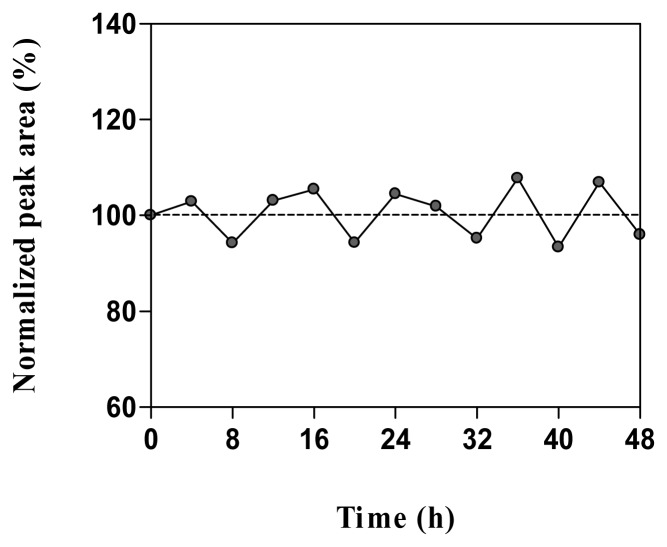
Normalized peak area obtained for calibration samples containing 0.3 nM acetylcholine in microdialysis matrix stored in the autosampler at 4°C.

Calibration samples containing acetylcholine in water or quality control samples in microdialysis matrix were stable for at least 48 h when stored at 4°C in the autosampler (Figure 5). After 3 months storage at -20°C no detectable concentration of acetylcholine was found in the microdialysis matrix. However, acetylcholine was previously reported to be stable for up to 2 months in a stock solution in water stored at 4°C [38] and in microdialysis samples stored at -85°C [18].

### 2.6. Method robustness and system suitability

**Figure 6.**
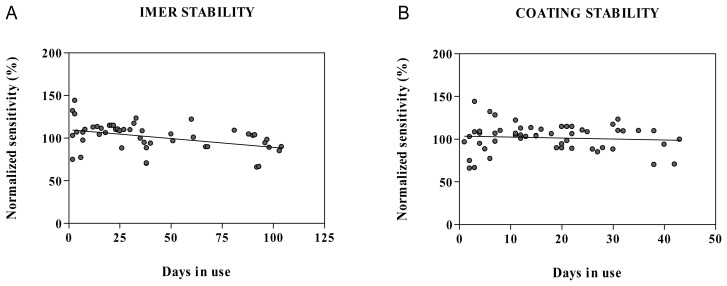
Normalized response for acetylcholine calibration standards (10 nM) as a function of **(A)** the number of days in use of the immobilized enzyme reactor or **(B)** the horseradish peroxidase redoxpolymer coating. The data obtained for five different enzyme reactors and 6 different enzyme coatings were pooled.

After the initial stabilization period of approximately five days, retention of acetylcholine on the chromatographic system and resolution from interfering matrix components was stable up for to four months of intensive use and reproducible for different batches of the chromatographic column (Figure 2B). However, frequent replacement of the inline filter was necessary. The observed loss of acetylcholine retention was associated with a decrease in the width of the injection peak. In the present study, the enzymatic stability was investigated by pooling the response for acetylcholine calibration standards for different enzyme reactors and cell coatings. A limited correlation was found between the sensitivity and the age of the enzyme reactor (r^2^=0.17) with an average drop in sensitivity of 0.20 ± 0.06 % per day (Figure 6A). No correlation was found between the sensitivity and the age of the enzyme coating (r^2^=0.01) with an average drop in sensitivity of 0.12 ± 0.18 % per day (Figure 6B). This suggests that the stability of the enzyme reactor rather than the cell coating is detrimental for the acetylcholine response. A rapid decrease in sensitivity was previously reported in an amperometric system with acetylcholine esterase and choline oxidase reactor and horseradish peroxidase osmium-redoxpolymer cell coating [22] whereas others reported a stable response over time when using a similar enzyme coated amperometric cell [12]. Differences in the coating protocol may account for the enhanced stability. Rather than a progressive decrease in sensitivity, a sudden deterioration was observed in the present study, necessitating replacement of the enzyme reactor every 2-3 months and coating of the cell every 1-2 months. Therefore, we suggest system suitability testing by injecting five replicate calibration standards at LLOQ level. The RSD for the precision should be less than 20% and the S/N ratio equal to or higher than 5.

### 2.7. Analysis of microdialysis samples

Recovery of acetylcholine is a function of the dimensions of the microdialysis membrane, the properties of the microdialysis membrane and the flow rate of the perfusion liquid [9, 39]. In the present study, microdialysis probes with a 4 mm polyethersulphon membrane (MAB6.14.4) were perfused at a flow rate of 1.5 μL/min. The mean ± SD concentration of acetylcholine in hippocampal microdialysis samples obtained from 32 rats under baseline conditions was 0.40 ± 0.12 nM. In hippocampal microdialysis samples from four rats the acetylcholine concentration was below the LLOQ and in samples from two rat's acetylcholine could not be detected. A typical chromatogram obtained for a microdialysis sample is shown in Figure 4D. Others similarly reported baseline acetylcholine concentration in hippocampal microdialysis samples in the range 0.1 – 1.6 nM [14, 22, 39]. Using the zero net-flux method, the baseline extracellular acetylcholine concentration in the hippocampus was estimated around 4 nM [39]. This suggests that in the present study the *in vivo* recovery for acetylcholine in microdialysis samples was approximately 10%. Further optimization of the microdialysis parameters may improve the probe recovery and thus the concentration of acetylcholine in the obtained microdialysates.

## 3. Experimental Section

### 3.1. Chemicals

Acetylcholine chloride, choline chloride, 1-hexanesulphonic acid sodium salt and 1-octanesulphonic acid sodium salt were purchased from Sigma-Aldrich Chemie (Steinheim, Germany). Tetramethylammonium bromide, Na_2_EDTA·2H_2_O, NaH_2_PO_4_·12H_2_0, NaCl and KCl were bought from Merck (Darmstadt, Germany) and CaCl_2_·6H_2_O from Fluka (Buchs, Switzerland). All the chemicals used were of analytical grade. Water was purified using a Seralpur Pro 90 CN purification system (Seral, Ransbach, Germany).

### 3.2. Liquid chromatography equipment and conditions

The liquid chromatography system consisted of a Gilson 307 pump (Gilson International, The Hague, The Netherlands) with a Gilson 805 manometric module and a Gilson 231 XL injector with a Gilson 832 temperature regulator maintaining sample temperature at 4°C. Injections of 20 μL were made in an internal loop mode on a 30 μL injection loop. The needle and loop wash solvent was water with 0.5% v/v of the microbiocide ProClin 150 (1%, Bioanalytical Systems, West Lafayette, USA). The mobile phase was degassed with an external Degasys DG-1210 vacuum degasser (Dionex, Amsterdam, The Netherlands) and pumped at a flow rate of 0.075 mL/min. A 0.5 μm inline filter (Alltech, Deinze, Belgium) was placed after the injector. The separation of cholines was obtained by isocratic reversed phase ion-pair chromatography on a Shiseido Capcell Pak C18 AQ with 150 mm column length, 1 mm internal diameter and 5 μm particle size (Analis, Gent, Belgium). The mobile phase was a 50 mM phosphate buffer at pH 6.5, containing 1 mM sodium 1-octanesulphonate, 2 mM tetramethylammonium bromide, 0.5 mM Na_2_EDTA·2H_2_O and 0.5% v/v ProClin 150 in water. The mobile phase was filtered before use over a Porafil 0.2 μm membrane filter (Macherey-Nagel, Düren, Germany). The chromatographic setup was maintained at an ambient temperature of 20°C. When the system was not in use the mobile phase was recycled at a flow rate of 0.02 mL/min.

### 3.3. Enzyme reactors and enzyme-modified amperometric detection

A postcolumn 50×1 mm acetylcholine oxidase and choline esterase IMER (Bioanalytical Systems) was used to convert acetylcholine into hydrogen peroxide. A horseradish peroxidase osmium-redoxpolymer (Bioanalytical Systems) coated cross-flow glassy carbon electrode with 2 mm surface diameter (Bioanalytical Systems) was used to detect hydrogen peroxide. The electrode was attached to an LC-4C amperometric detector (Bioanalytical Systems) operated in reductive mode at +100 mV, 0.2 nA/V sensitivity and 0.1 Hz signal filtration. The enzyme reactors and the amperometric detector were maintained at an ambient temperature of 20°C. Before each new coating, the electrode surface was cleaned with water, rinsed with acetone and dried in a dust free box. The electrode surface was coated with 0.5 μl of a coating surfactant (Bioanalytical Systems) to facilitate spreading and adhesion of the osmium-peroxidase redoxpolymer to the electrode surface. During method development a single and a double enzyme coating protocol were compared. After letting the surfactant dry for 15 min, a layer of 0.5 μL horseradish peroxidase osmium-redoxpolymer was applied onto the electrode surface. After 15 minutes drying, a second layer of 0.5 μL horseradish peroxidase osmium-redoxpolymer was applied onto the electrode surface. The redoxpolymer was left to harden for 4 h before use.

### 3.4. Microdialysis equipment and sampling

Protocols for the experiments on freely moving rats were carried out according to the European directive 86/609/EEC with the corresponding national guidelines and approved by the Ethical Committee for Animal Experimentation of the Faculty of Medicine and Pharmacy of the Vrije Universiteit Brussel. Male Wistar rats were obtained from Charles-River Laboratories (L'Arbresle, France) and weighed 275 g to 320 g at the time of surgery. General anaesthesia was induced by an intraperitoneal injection of 60 mg/kg ketamine (Ketamine 1000 mg/ml Ceva, Ceva Sante Animale, Brussels, Belgium) and 5 mg/kg diazepam (Valium 10 mg/2mL, Roche, Brussels, Belgium). Body temperature was maintained at 37°C during surgery using a heating pad. Intracerebral guide cannulae (MAB2/6/9.14.IC, Microbiotech/se, Stockholm, Sweden) with replaceable dummy cannulae were stereotaxically implanted above the hippocampus 4.6 mm lateral from midline, 5.6 mm caudal from bregma and 3.6 mm ventral from dura using flat skull coordinates (Paxinos and Watson, 1986). The guide cannulae were fixed to the skull with two achor screws (CMA/Microdialysis, Stockholm, Sweden) and carboxylate dental cement (Durelon, 3M ESPE, Seefeld, Germany). Post-operative analgesia was provided with an intraperitoneal injection of 4 mg/kg ketoprofen (Ketofen 1%, Merial, Brussels, Belgium). The dummy cannulae were replaced by concentric microdialysis probes with 4 mm membrane length (MAB6.14.4; Microbiotech/se) immediately after surgery and the animals were allowed to recover overnight in a microdialysis bowl with free access to food and water. One hour before the start of the experiment the microdialysis probe was perfused at a flow rate of 1.5 μL/min with a modified Ringer's solution containing 147 mM NaCl, 4 mM KCl and 2.3 mM CaCl_2_ in water. Microdialysis matrix for the preparation of quality control samples were continuously collected from 6 different rats into 2 mL polypropylene tubes (Brand, Wertheim, Germany) and stored at -20°C until use. Microdialysis samples for routine analysis were collected every 20 min into 0.7 mL polypropylene tubes (Gilson International) and stored at 4°C until analyzed within 48 h.

### 3.5. Preparation of stock solutions, calibration standards and quality control samples

Stock solutions of acetylcholine chloride (1 M) and choline chloride (1 M) were prepared daily and refrigerated at 4°C. The analytes were weighed using a calibrated balance (model AG204, Mettler-Toledo, Greifensee, Switzerland). Acetylcholine calibration standards and quality control samples were prepared by diluting the stock solution in water or microdialysis matrix respectively. Microdialysis matrices from two rats were thawed and pooled on each day of validation. All dilutions were made using calibrated micropipettes (Eppendorf Reference, Eppendorf, Germany) in 0.7 mL polypropylene tubes (Gilson International). Samples for routine analysis were directly collected into 0.7 mL polypropylene tubes (Gilson International) and analyzed without further manipulation.

### 3.6. Statistical analysis

Statistics were performed with the GraphPad 4 software. The linear regression method was used to calculate the calibration curves. Linearity of the calibration curves was evaluated using an analysis of variance lack of fit test. Differences in the curves obtained with calibration standards prepared in water and quality control samples prepared in microdialysis matrix were analyzed using a two-way analysis of variance. The repeatability and inter-day precision of the method were calculated over three days of validation using one-way analysis of variance. The accuracy was calculated as the average accuracy obtained over three consecutive days of validation. Values for p < 0.05 were considered significant.

## 4. Conclusions

To our knowledge this is the first report in which a liquid chromatography system with amperometric detection is validated in the concentration range required for acetylcholine determination in rat brain microdialysis samples. The present assay balanced sensitivity to enzymatic and chromatographic stability and was precise and accurate for the quantification of acetylcholine in microdialysis samples without the use of cholinesterase inhibitors. The mobile phase with pH to 6.5 allowed for adequate resolution of acetylcholine from choline and other matrix components for up to 4 months on a microbore polymer-coated silica column with high endcapping and low octadecyl binding-density. However, the present method was unable to resolve choline from the injection peak. The acetylcholine esterase and choline oxidase IMER was stable up to 2-3 months and the enzyme coating was stable up to 1-2 months. With adequate maintenance this system allows the routine analysis of acetylcholine in microdialysis samples.

## Figures and Tables

**Table 1.** Precision and accuracy of the assay for determination of acetylcholine in rat brain microdialysates (five replicates per day on three separate days).

**Added Concentration (nM)**	**Mean ± SD measured concentration (nM)**			**Repeatability (RSD %)**	**Inter-day precision (RSD %)**	**Accuracy (%)**

	**Day 1**	**Day 2**	**Day 3**			

0.30	0.30 ± 0.04	0.32 ± 0.05	0.32 ± 0.05	15.7	8.6	103.6
1.00	1.02 ± 0.07	0.98 ± 0.11	1.03 ± 0.05	7.4	5.1	100.9
3.00	2.95 ± 0.06	3.03 ± 0.27	2.88 ± 0.07	4.4	5.1	98.4
10.0	9.60 ± 0.37	9.79 ± 0.44	9.96 ± 0.50	4.4	4.0	97.8
